# Carbonic Anhydrase III Has Potential as a Biomarker for Experimental Colitis and Functions as an Immune Regulator by Inhibiting Inflammatory Cytokine Secretion

**DOI:** 10.3390/biology11040494

**Published:** 2022-03-23

**Authors:** Kohki Okada, Masaki Ikemoto

**Affiliations:** 1 Department of Medical Technology and Sciences, Faculty of Health Sciences, Kyoto Tachibana University, Kyoto 607-8175, Japan; 2 Faculty of Bioscience, Nagahama Institute of Bio-Science and Technology, Nagahama 526-0829, Japan; m_ikemoto@nagahama-i-bio.ac.jp

**Keywords:** carbonic anhydrase III, inflammatory cytokines, macrophage, ulcerative colitis

## Abstract

**Simple Summary:**

The mechanism underlying the onset of ulcerative colitis (UC) has not yet been elucidated in detail. Unknown components in colorectal tissue may be important risk factors to elucidate the cause of UC; however, they have not been highlighted as targets. To identify key factors, rats with dextran sulfate sodium-induced experimental colitis were used. The level of carbonic anhydrase III was significantly decreased in both the serum and colon tissues of these UC rats. Upon stimulation of peritoneal macrophages (MΦ) with lipopolysaccharide, the intracellular concentration of carbonic anhydrase III significantly decreased, while the secretion of pro-inflammatory cytokines from MΦ treated with an anti-carbonic anhydrase III antibody was negatively regulated. In conclusion, carbonic anhydrase III may be a novel regulator of experimental colitis in rats.

**Abstract:**

Ulcerative colitis (UC) is characterized by chronic inflammation of the large intestine, repeated remissions, and symptom relapses. Although unknown components in colonic regions are deeply involved in the pathogenesis of UC, the causes of UC development and aggravation have not yet been elucidated in detail. To identify key factors, we investigated the changes in protein components in the large intestine of rats with dextran sulfate sodium-induced experimental colitis (UCR). The components that differed in their concentration between normal rats (WT) and UCR were carefully investigated by electrophoretic separation and mass spectrometry. Based on these results, seven proteins with different expression levels between the WT and UCR were observed. Among them, we focused on carbonic anhydrase III (CA-III) in the pathogenesis of UC. CA-III concentrations in the colon tissue and serum were quantitatively measured using an enzyme-linked immunosorbent assay (ELISA) and real-time PCR, and the levels significantly decreased in both the colon tissue and serum of UCR with the aggravation of experimental UC. In an *in vitro* assay, CA-III function in peritoneal macrophages (MΦ) from rats was investigated. Upon stimulation of MΦ with lipopolysaccharide (LPS), the CA-III concentration significantly decreased in the cytoplasm of these cells. MΦ treated with an anti-CAIII antibody followed by stimulation with LPS actively secreted inflammatory cytokines, particularly interleukin-6 and tumor necrosis factor-α. Therefore, CA-III in MΦ appears to be an immune regulator that suppresses the secretion of inflammatory cytokines.

## 1. Introduction

Ulcerative colitis (UC) is classified as an inflammatory bowel disease (IBD) and is characterized by chronic and recurrent inflammation in the large intestine, particularly in the rectal region [1,2]. A large number of patients with UC have been confirmed in Europe and North America, and the incidence of UC has been increasing in Asia, South America, and Africa [3]. Thus, the marked increase in UC is currently an international problem. Therefore, a comprehensive understanding of the pathogenesis of this disease is needed. However, despite extensive investigations by many scientists, the mechanism underlying the onset of UC has not yet been elucidated in detail. Some factors that contribute to the onset of UC, such as environmental, genetic, and immunological factors, have been identified [4], of which abnormalities in antigen-presenting cells, such as macrophages, to commensal bacteria in the colon have been widely noticed [5]. Some immunosuppressive agents, such as tacrolimus and cyclosporine, are currently used to treat UC because they are clinically effective in reducing immune cell abnormalities [6]. However, these drugs may also suppress the immune system of healthy individuals. Therefore, the side effects caused by these drugs are always a concern during the administration period. Furthermore, the lack of therapeutic targets for the treatment of UC has hindered the establishment of effective therapies for this disease.

The S100 proteins (S100A8, S100A9, and S100A8/A9), the levels of which fluctuate in the large intestine, have been the focus of research on the pathogenesis of UC. They have been strongly implicated in the aggravation of UC via toll-like receptor 4 or receptor for advanced glycation end products [7,8,9,10,11]. We previously reported that S100A9 was predominantly expressed in the macrophages of rectal tissues in rats with experimental UC (UCR) induced by 5% dextran sulfate sodium (DSS) [12]. We also confirmed that S100A8 negatively regulates the onset of UC in originally established transgenic rats (Tg-S100A8) that abundantly express S100A8 in many organs, including the intestinal tract [13]. Furthermore, stool concentrations of S100A8/A9 (calprotectin) correlated with disease activity index (DAI) scores in patients with UC [14,15]. In our clinical study, serum concentrations of calprotectin did not correlate with DAI scores in patients with UC [16]; however, we subsequently demonstrated that serum complement C3 and α_2_-macrogulobulin concentrations correlated with DAI scores in these patients [17]. Leucine-rich alpha-2-glycoprotein and prostaglandin E-major urinary metabolite are also widely known as new biomarkers for clinically evaluating the severity of UC [18,19]. Although these biomarkers may reflect the disease activity of UC to some extent, they have not been shown to be useful biomarkers for therapeutic targets.

Many researchers have suggested that inflammatory cytokines and CXCL chemokines are useful biomarkers in UC, with inhibition of their secretion potentially contributing to the control of inflammation [20,21]. Activation of the signal transduction and activator of transcription 3 (STAT3) pathway in intestinal epithelial cells by interleukin (IL)-6 may result in the onset of UC [22]. However, these proteins do not specifically fluctuate in the serum, including in the inflammatory region, in patients with UC. To identify novel biomarkers and therapeutic targets for UC, it is necessary to specify components in the large intestine, particularly the rectal region, using animal models. The aim of the present study was to identify proteins involved in the pathogenesis and aggravation of experimental UC. In the in vitro study, we also examined the immune functions of proteins identified using peritoneal macrophages (MΦ) from rats treated with or without lipopolysaccharide (LPS).

## 2. Materials and Methods

### 2.1. Ethics Statement

The animal experiments complied with ARRIVE guidelines and were approved by the Animal Experiment Committee of Kyoto Tachibana University (permission number: 20-03).

### 2.2. Animals

Wild type Slc:Wistar rats (WT; male, 9 weeks old, 220–250 g/rat) were purchased from Shimizu Laboratory Supplies Co., Ltd. (Kyoto, Japan). They were housed for approximately one week prior to the experiments and were allowed free access to food and water.

### 2.3. Reagents

Pre-packed disposable PD-10 columns were purchased from Global Life Science Technologies Japan Co., Ltd. (Tokyo, Japan). Anti-CA3/carbonic anhydrase III mouse monoclonal antibody (anti-CAIII Ab) was obtained from Funakoshi Co., Ltd. (Tokyo, Japan). Anti-mouse IgG (goat)-horseradish peroxidase (HRP) or -fluorescein 5-isothiocyanate (FITC) conjugate and streptavidin (STA)-tetramethylrhodamine (TRITC) were purchased from Abcam (Cambridge, UK). The CA3/Carbonic anhydrase III enzyme-linked immunosorbent assay (ELISA) kit and PRO-PREP^TM^ Protein Extraction Solution (Cell/Tissue) were purchased from Cosmo Bio Co., Ltd. (Tokyo, Japan). The DSS salt (MW: 36000–50000) was purchased from Wako Pure Chemical Industries, Ltd. (Tokyo, Japan). Clinical thioglycollate medium (E-MC17) was obtained from Eiken Chemical Co., Ltd. (Tokyo, Japan). LPS from *Escherichia coli* was purchased from Sigma-Aldrich Co., LLC (Tokyo, Japan). VECTASHIELD mounting medium containing 4′,6-diamidino-2-phenylindole (DAPI) was obtained from Vector Inc. (Burlingame, CA, USA). *N*-Hydroxysuccinimidobiotin (EZ-Link^TM^ NHS-Biotin), TRIzol^TM^ reagent, SuperScript^TM^ II Reverse Transcriptase, PowerUp SYBR Green Master Mix, and all primers were purchased from Thermo Fisher Scientific (Waltham, MA, USA). All other reagents were obtained from Wakenyaku Co., Ltd. (Kyoto, Japan), Nacalai Tesque Co., Ltd. (Kyoto, Japan), and Bio-Rad Laboratories, Inc. (Hercules, CA, USA).

### 2.4. Protocol for Animal Experiments 

**Figure 1:**
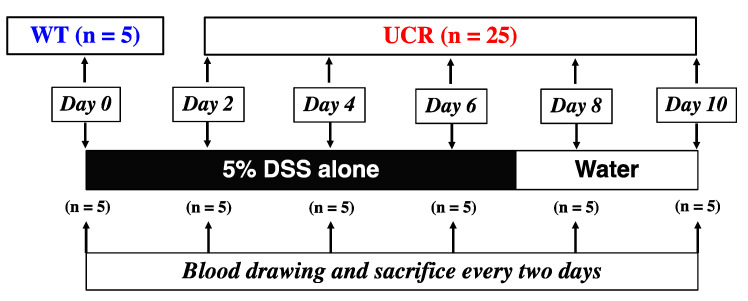
Protocol of animal experiments. WT: normal wild type Slc:Wistar rats (n = 5) were euthanized as negative controls (Day 0). UCR: Slc:Wistar rats were treated with 5% dextran sulfate sodium (DSS) as experimental ulcerative colitis model rats (n = 25). Five rats were euthanized every two days (Days 2–10), and their large intestines and blood were collected.

To generate experimental UC model rats (UCR, n = 25), WT rats were orally administered 5% DSS in distilled water (DW) for 10 days. During this period, the body weight of each rat was measured every morning, and the DAI scores were evaluated [23] based on the criteria shown in Table 1. Five rats were analyzed every two days from Day 2 to Day 10 as follows: blood samples (3 mL/rat) were immediately collected from the heart under anesthesia. After euthanasia, the large colon of each rat was quickly removed and its length was measured. The large colon was divided into three segments (rectum, middle colon, and proximal colon). Specimens from each segment were fixed in 10% formalin/0.1 M phosphate buffer (pH 7.4) for histological assessments and then embedded in paraffin. Protein and mRNA in the residual unfixed tissue of each segment were extracted as described below. Normal WT rats (n = 5) were used as negative controls (Day 0), and a similar procedure was performed. The protocol for the animal experiments is summarized in Figure 1.

### 2.5. Sample Preparation for the Protein Assay

The three tissue segments (rectum, middle colon, and proximal colon) collected from each rat were independently weighed. Three hundred micrograms of each sample was incubated in 0.5 mL PRO-PREP^TM^ Protein Extraction Solution (Cell/Tissue) for 10 min and then centrifuged at 12,000 rpm at 4 °C for 10 min. The resultant supernatants were transferred into 1.5 mL polycarbonate tubes and stored at −80 °C until use.

### 2.6. Sample Preparation for the mRNA Assay

Three hundred micrograms of the three tissue segments was independently incubated in 1.0 mL TRIzol^TM^ reagent for 10 min and then centrifuged at 12,000 rpm at 4 °C for 15 min. The mRNA from each sample was extracted according to the manufacturer’s protocol and then treated with 8 M LiCl to avoid the influence of DSS on RNA reverse transcription. cDNA was synthesized from the mRNA using SuperScript^TM^ II Reverse Transcriptase, as described in the instruction manual.

### 2.7. Gel Filtration Chromatography (GFC)

Protein extracts collected from the rectum of the UCR on Day 10 (n = 5, 0.2 mL each, total volume of 1.0 mL) were mixed in a 1.5 mL polycarbonate tube. The mixture of protein extracts was subsequently added to a PD-10 column for GFC. The column was thoroughly washed with 0.3 mL of 10 mM Tris-HCl buffer (pH 7.4) and 0.9% NaCl (buffer A), and 0.3 mL samples of each wash fraction were collected in microtubes (1.5 mL). This procedure was repeated until 18 fractions were collected in the microtubes. The same experiment was conducted using protein extracts from the rectum of WT rats (n = 5) as a negative control.

### 2.8. Sodium Dodecyl Sulfate-Polyacrylamide Gel Electrophoresis (SDS-PAGE)

After GFC, the proteins in each fraction were separated by SDS-PAGE in the presence of 2-mercaptoethanol, as previously described [24]. The concentration of all polyacrylamide gels was 12.5%. Coomassie Brilliant Blue staining was performed to visualize the protein bands in each gel.

### 2.9. Matrix-Assisted Laser Desorption Ionization Time-of-Flight (MALDI-TOF) Mass Spectrometry 

After SDS-PAGE, some protein bands were excised and delivered to the MALDI-TOF mass spectrometry accession service (Cosmo Bio Co., Ltd.) for analyses. Briefly, a single protein band was digested with trypsin, and peptide mass fingerprint analysis was conducted using Microflex LRF 20 (Bruker Daltonics Corp., Billerica, MA, USA). The fragments of protein components were identified using a Mascot search of the National Center for Biotechnology Information (NCBI) database. Mascot scores >67 were considered statistically significant.

### 2.10. ELISA

Carbonic anhydrase III (CA-III) in each sample was measured using an ELISA kit, according to the manufacturer’s instructions. The absorbance of the color reaction was measured at 450 nm using a microplate reader (iMark™; Bio-Rad Laboratories, Inc., Hercules, CA, USA).

### 2.11. Microscopic Observation of the Rectal Tissue of Each Rat

We microscopically observed rectal tissues with severe inflammation in the DSS-induced UC rats. Three micrometer thick tissue sections obtained from the rectal tissues of UCR and WT rats were stained with hematoxylin and eosin (H&E). Tissue damage was histologically evaluated and assessed based on the H&E staining, and the extent of damage was scored based on the criteria shown in Table 2 [23,25]. The expression of CA-III in tissues and MΦ was detected by immunohistochemical staining with diaminobenzidine (DAB) and fluorescent immunochemical staining (FICS), respectively, using the anti-CAIII Ab, according to previously described methods [13]. Microscopic images were obtained using a BIOREVO BZ-9000 microscope (Keyence Co. Ltd., Osaka, Japan). 

### 2.12. Western Blotting

After SDS-PAGE, the proteins were transferred to nitrocellulose membranes using a Trans-Blot Turbo (Bio-Rad Laboratories, Inc.). After the membranes were blocked with Blocking One (Nacalai Tesque Co., Ltd.), they were incubated at 4 °C for 1 h with 2 μg/mL of anti-CAIII Ab. The membranes were then washed three times for 5 min with buffer A, twice with buffer A/0.1% Tween 20, and once with buffer A before being incubated with 2 μg/mL rabbit anti-mouse IgG H&L (HRP) at room temperature for 1 h. After the membranes were washed, antibody-bound proteins were detected using the ChemiDoc^TM^ XRS Plus Imaging System and Clarity Western ECL substrate (Bio-Rad Laboratories, Inc.).

### 2.13. Real-Time PCR

Real-time PCR was performed using the StepOnePlus Real-Time PCR System (Thermo Fisher Scientific) as previously described [11]. The primers used were as follows: CA-III forward, 5′-GATAGGACGGGAGAAAGGCG-3′, CA-III reverse, 5′-GAGCCTCCTTGCCCTTAGTC-3′ (77 bp), IL-1β forward, 5′-CACCTCTCAAGGAGAGCACAGA-3′, IL-1β reverse, 5′-CACCTCTCAAGGAGAGCACAGA-3′ (81 bp); IL-6 forward, 5′-ATATGTTCTCAGGGAGATCTTGGAA-3′, IL-6 reverse, 5′-GTGCATCATCGCTGTTCATACA-3′ (80 bp); IL-10 forward, 5′-GCCAAGCCTTGTCAGAAATGA-3′, IL-10 reverse, 5′-TTTCTGGGCCATGGTTCCTCT-3′ (75 bp); tumor necrosis factor (TNF)-α forward, 5′-GTGATCGGTCCCAACAAGGA-3′, TNF-α reverse, 5′-AGGGTCTGGGCCATGGAA-3′ (71 bp); transforming growth factor (TGF)-β forward, 5′-ACCTGCAAGACCATCGACATG-3′, TGF-β reverse, 5′-CGAGCCTTAGTTTGGACAGGAT-3′ (85 bp); and β-actin forward, 5′-TGTGTTGTCCCTGTATGCCTCTG-3′, β-actin reverse, 5′-ATAGATGGGCACATGGTGGGTG-3′ (85 bp).

### 2.14. Isolation of MΦ from Rats

MΦ were isolated from WT rats as previously described [11]. Briefly, MΦ were induced by intraperitoneal injection of sterilized 4% thioglycollate/DW (10 mL). After three days, the MΦ were collected in a plastic tube (50 mL) using 50 mM sterilized phosphate buffer solution (pH 7.4) and 0.9% NaCl (buffer B). The tube was then centrifuged at 3500 rpm at 4 °C for 5 min, and the supernatant was discarded. The cells collected in the tube were suspended in 17 mM Tris-HCl (pH 7.2) containing 0.83% NH_4_Cl and incubated at 37 °C for 10 min to induce hemolysis of the contaminating erythrocytes. After centrifugation as above, the supernatant was discarded, and the pelleted cells were suspended in RPMI-1640 culture medium containing 10% fetal bovine serum (Biological Industries, Kibbutz Beit-Haemek, Israel; medium A). A total of 2 × 10^6^ cells were plated in each well of a six well plate, and an appropriate volume of medium A was added. The cells were then incubated at 37 °C for 2 h in 5% CO_2_. After incubation, the non-adherent cells in each well were removed by washing three times with buffer B. The adherent cells were maintained in the same medium at 37 °C in 5% CO_2_ until use.

### 2.15. Stimulation of MΦ from Rats with LPS

After thorough washing with buffer B, the MΦ were stimulated with LPS (1 μg/mL) in medium A for 0.5, 1, or 2 h. After washing three times with buffer B, the cells were subjected to FICS or collected with 0.2 mL of buffer A in a polycarbonate tube (1.5 mL). Protein and mRNA were extracted from the collected cells using PRO-PREP^TM^ Protein Extraction Solution and TRIzol™ reagent, respectively. The intracellular expression of CA-III was evaluated using western blotting, real-time PCR, and FICS.

### 2.16. Statistical Analysis 

Pairwise comparisons with controls were performed using non-parametric tests. Significant differences between the groups were identified using the Mann–Whitney U test. Data are shown as the mean ± standard deviation (SD). Statistical significance was set at *p* < 0.05. The relationship between the two groups in each case was assessed by Spearman’s test using the statistical software “Easy R version 1.54” [26]. A correlation coefficient (R-value) of between 0.5 and 1.0 indicated a good correlation. 

## 3. Results

### 3.1. Microscopic Observations and Evaluation of Inflammation in the Rectal Region

**Figure 2:**
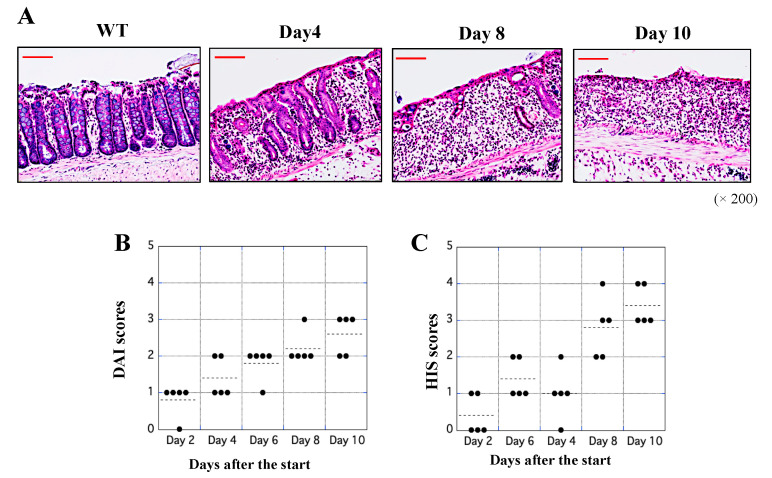
Evaluation of disease severity. H&E staining was performed to visualize histological changes in the rectal tissues of WT and UCR. (**A**) Microscopic images of the rectal tissues of WT and UCR on Days 4, 8, and 10 after the start of the experiments. High power magnification was used for all panels (×200). Red scale bar = 50 μm. (**B,C**) DAI and HIS scores on Days 2, 4, 6, 8, and 10 after the start of the experiments were assessed based on the criteria of Table 1 and Table 2. Dotted points indicate average measurements on each day. WT: wild type Slc:Wistar rats; UCR: Slc:Wistar rats treated with 5% dextran sulfate sodium; DAI: disease activity index; HIS: histological.

H&E staining was performed to visualize the tissue damage and immune cells in the rectal region. Comparisons with WT tissue revealed that the epithelial structure of the rectal region of the UCR was damaged, and a large number of immune cells had infiltrated the tissue during the course of the experimental period (Figure 2A). We evaluated the DAI scores based on the criteria listed in Table 1, as previously described [23]. The DAI scores increased in the UCR group as physical symptoms gradually worsened (Figure 2B). HIS scores were assessed based on the criteria shown in Table 2, as previously described [25]. Based on the results of H&E staining, severe inflammation of the rectal region in UCR was observed. This observation reflected high HIS scores, particularly on Days 8 and 10 (Figure 2C).

### 3.2. Comparison of Components in the Rectal Region between WT and UCR

**Figure 3:**
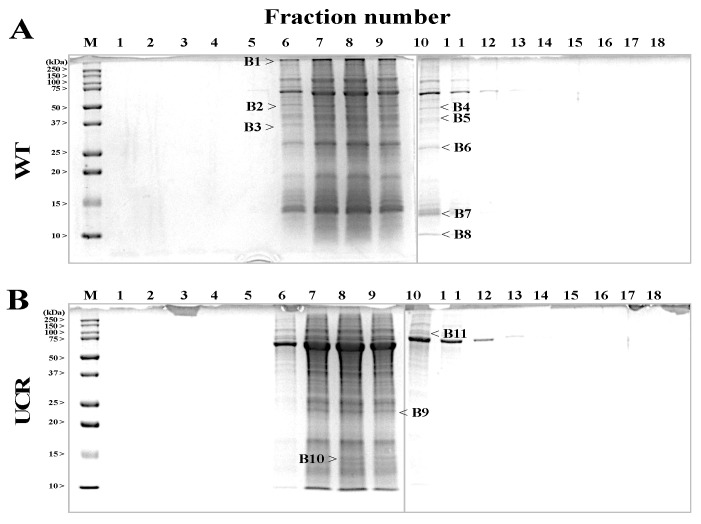
Fractionation and separation of rectal proteins in WT and UCR. The rectal proteins of WT and UCR were fractionated using gel filtration columns. The protocol is described in Section 2.7 in detail. Panels (**A**,**B**) show protein bands separated by SDS-PAGE from each fraction of WT and UCR, respectively (Figures S1 and S2). Lane M is the molecular weight markers, and lanes 1 to 18 are the eighteen fractions after gel filtration chromatography. The protein bands of B1 to B11 (black arrows, > or <) were selected and subjected to mass spectrometry (MALDI-TOF). WT: Wild type Slc:Wistar rats; UCR: Slc:Wistar rats treated with 5% dextran sulfate sodium.

Proteins in the rectal region of WT and UCR were fractionated by GFC according to our original method (see Section 2.7). After several trials, 18 fractions were obtained from the protein samples derived from the WT or UCR. These fractions were further separated by SDS-PAGE. Although the separation performance of GFC was moderate, we carefully examined each protein band in the gel and selected 11 bands (B1–B11) with different densities between WT and UCR (Figure 3, Figures S1 and S2). Among these protein bands, the density of B1 to B8 in UCR was lower than that in WT, whereas the density of B9 to B11 in WT was higher than that in UCR. Therefore, these 11 protein bands were subjected to mass spectrometry. 

### 3.3. Identification of Rectal Proteins Using Mass Spectrometry 

**Figure 4:**
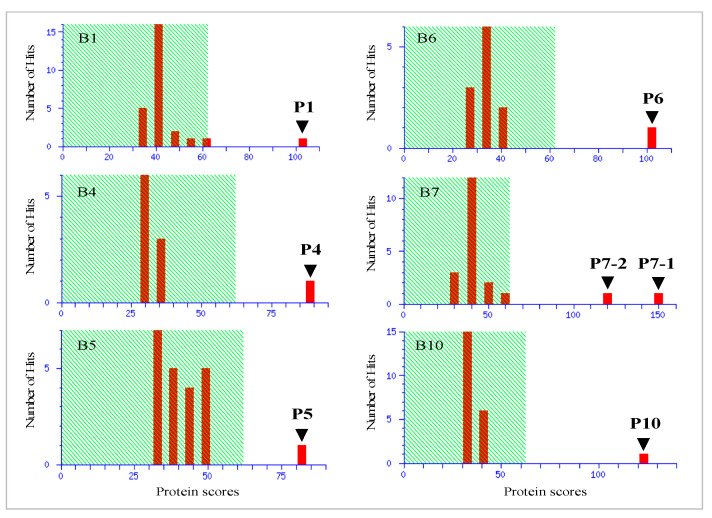
Histograms of identified protein components by mass spectrometry. The results of score histograms of six proteins (B1, B4, B5, B6, B7, and B10) were successfully obtained by mass spectrometry (MALDI-TOF) of 11 proteins (B1–B11). Seven components (P1, P4, P5, P6, P7-1, P7-2, and P10) were identified (Table 3) via the NCBI database. Protein scores > 67 are significant.

Apart from a few protein bands (B2, B3, B8, B9, and B11), six residual protein bands yielded good scores (>67). Consequently, seven proteins (P1, P4, P5, P6, P7-1, P7-2, and P10) were identified based on information available from the NCBI database (Figure 4). The results are summarized in Table 3. 

### 3.4. Determination of CA-III Levels in the Rectal Region and Serum

**Figure 5:**
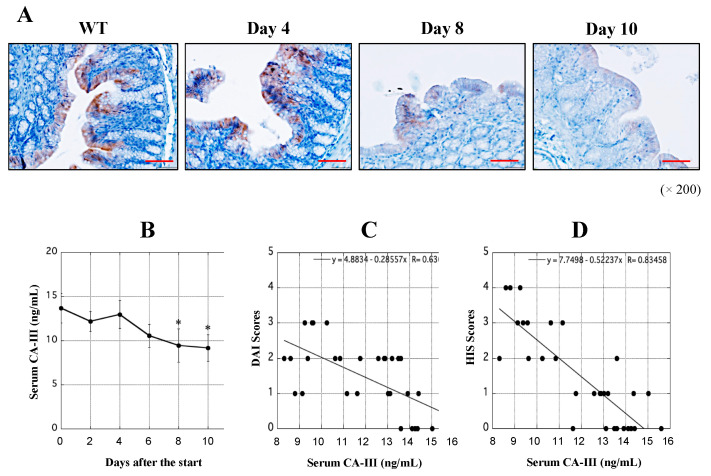
Clinical evaluation of CA-III in rectal tissues and serum of WT and UCR. (**A**) Microscopic images of immunohistochemical staining of CA-III in rectal tissue of WT and UCR on Days 4, 8, and 10 after the start of the experiments. High power magnification was used for all panels (×200). Red scale bar = 50 μm. (**B**) Serum concentration of CA-III (ng/mL) in WT (Day 0) and UCR (Days 2–10) was quantified by ELISA. X-axis indicates days after the start of the experiment, while Y-axis shows the concentration of CA-III (ng/mL). Data are the mean ± SD. * *p* < 0.05. (**C**,**D**) Relationship between serum CA-III concentrations and DAI or HIS scores, respectively, was assessed using Spearman’s analysis. X-axis indicates the CA-III concentration (ng/mL), while Y-axis shows the value of DAI or HIS score. R-values between 0.5 and 1.0 are significant. WT, Wild type Slc:Wistar rats; UCR, Slc:Wistar rats treated with 5% dextran sulfate sodium; DAI, disease activity index; HIS, histological; CA-III, carbonic anhydrase III.

To observe differences in the extent of CA-III protein expression in the rectal region of the large colon of UCR, immunohistochemical staining was performed using a specific antibody for CA-III, in which DAB was used as a substrate for color development. CA-III was mainly expressed in the normal mucosal epithelium in the rectal tissue of WT, whereas the CA-III-positive region gradually shrunk in size and ultimately disappeared with the aggravation of UC (Figure 5A). ELISA showed that the serum concentrations of CA-III were significantly lower in UCR (Days 2–10) than in WT rats (Day 0), particularly on Days 8 and 10 (Figure 5B). Based on these results, the relationship between the serum concentrations of CA-III and the severity of experimental UC was evaluated. The serum concentrations of CA-III correlated with DAI scores (R = 0.637, Figure 5C) and HIS scores (R = 0.835, Figure 5D). These results indicate that the serum CA-III level is a useful biomarker for assessing the severity of experimental UC.

### 3.5. Evaluation of CA-III Expression in the Large Intestine 

**Figure 6:**
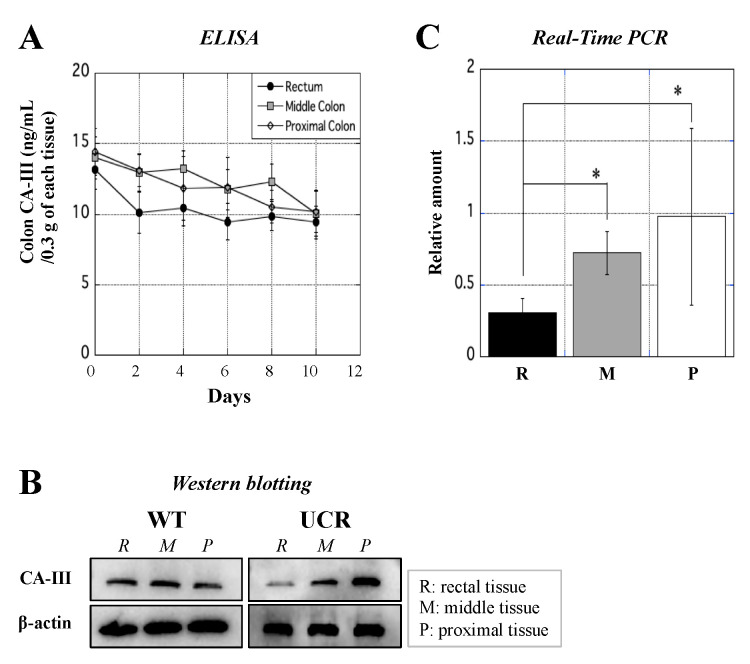
Measurement of CA-III concentrations in the colon of WT and UCR by ELISA, western blotting, and real-time PCR. (**A**) Concentration of CA-III (ng/mL/0.3 g of each tissue) in the large colon (rectum, middle colon, and proximal colon) of WT (Day 0) and UCR (Days 2–10) was measured by ELISA. X-axis indicates days after the start of the experiment, while Y-axis indicates the concentration of CA-III (ng/mL/0.3 g of each tissue) in each region. Data are the mean ± SD. (**B**) Western blotting was performed to detect CA-III in each colonic region (Figure S3). The upper and lower panels show the expression of CA-III and β-actin (internal control) protein, respectively, in the rectum (R), middle colon (M), and proximal colon (P) in WT (left panel) and UCR (right panel). (**C**) Real-time PCR shows the relative amount of CA-III mRNA in each colonic region of UCR against that of WT. X-axis indicates each region, while Y-axis indicates the relative amount of CA-III mRNA in each segment of UCR against that of WT. Data are the mean ± SD. * *p* < 0.05. WT: Wild type Slc:Wistar rats; UCR: Slc:Wistar rats treated with 5% dextran sulfate sodium; CA-III: carbonic anhydrase III.

ELISA, real-time PCR, and Western blotting were performed to investigate the expression of CA-III in more detail. ELISA showed that CA-III in the three colon fragments gradually decreased over the course of the experimental period (Figure 6A). In addition, Western blotting showed that CA-III levels in the rectal tissue of UCR significantly decreased on Day 10. The mRNA expression of CA-III in the UCR group on Day 10 was assessed by Western blotting (Figure 6B and Figure S3); it was markedly lower in the rectal tissue than in the other two segments by real-time PCR (Figure 6C).

### 3.6. Influence of LPS Stimulation on CA-III Expression in MΦ

**Figure 7:**
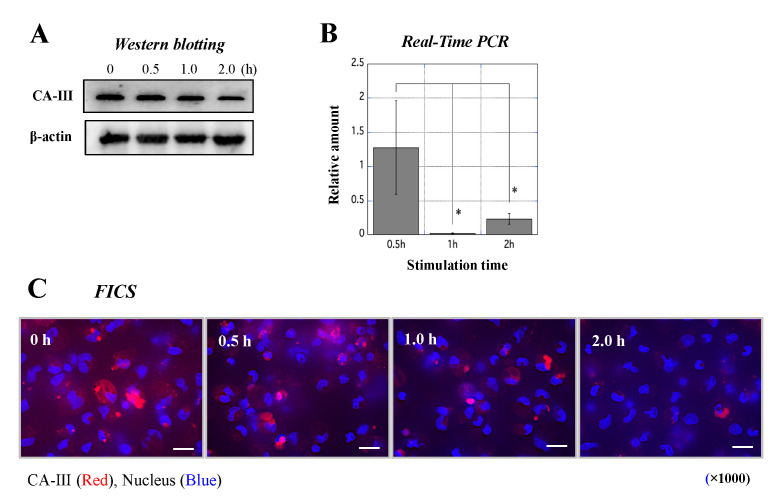
Measurement of CA-III expression in rat MΦ stimulated with LPS by western blotting, real-time PCR, and immunofluorescence. (**A**) Western blotting was performed to detect CA-III in MΦ stimulated with LPS (Figure S4). The upper and lower panels show CA-III and β-actin (internal control) expression, respectively, in MΦ stimulated with LPS for 0, 0.5, 1, and 2 h. (**B)** Real-time PCR was conducted to analyze the relative amount of mRNA in MΦ stimulated with LPS. The X-axis indicates the stimulation time (0.5, 1, and 2 h), while the Y-axis shows the relative amount of CA-III in stimulated MΦ relative to CA-III in unstimulated MΦ. Data are the mean ± SD. * *p* < 0.05. (**C**) Microscopic observations of CA-III expressed in MΦ stimulated with LPS for 0, 0.5, 1, and 2 h by immunofluorescence. CA-III (red) and nuclei (blue) were stained according to previously published methods [11]. High power magnification images are shown for all panels (×1000). White scale bar = 10 μm. CA-III: carbonic anhydrase III; LPS: lipopolysaccharide; MΦ: peritoneal macrophages; FICS: fluorescent immunochemical staining.

Throughout the course of the in vivo study, we speculated that CA-III plays a pivotal role in MΦ in the large colon tissues of rats with experimental UC. An in vitro study was conducted to test our hypothesis using MΦ derived from WT. The amount of CA-III in the MΦ gradually decreased after LPS stimulation (Figure 7A and Figure S4). Real-time PCR showed that upon stimulation of MΦ with LPS, CA-III mRNA levels significantly decreased in the cells (Figure 7B). Microscopic observations were performed to confirm CA-III expression in MΦ using immunofluorescence staining. CA-III was mainly expressed in the cytoplasm of normal MΦ but gradually disappeared from the cells after stimulation with LPS (Figure 7C).

### 3.7. Influence of Antibody Treatment on CA-III Levels in MΦ

**Figure 8:**
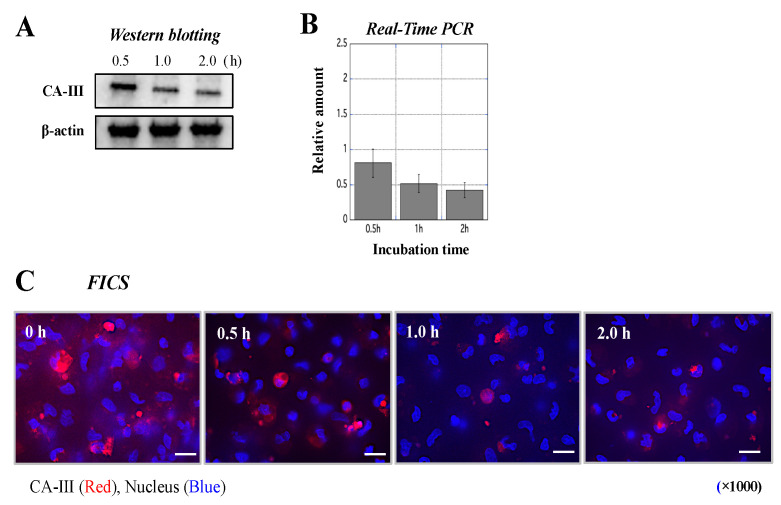
Measurement of CA-III expression in rat MΦ incubated with anti-CAIII Ab by western blotting, real-time PCR, and immunofluorescence. (**A)** Western blotting was performed to detect CA-III in MΦ incubated with anti-CAIII Ab (Figure S5). The upper and lower panels show CA-III and β-actin (internal control) expression, respectively, in MΦ incubated with anti-CAIII Ab for 0.5, 1, and 2 h. (**B**) Real-time PCR was performed to analyze the relative amount of mRNA in MΦ incubated with anti-CAIII Ab. The X-axis indicates the incubation time (0.5, 1, and 2 h), while the Y-axis shows the relative amount of CA-III in incubated MΦ relative to CA-III levels in non-incubated MΦ. Data are the mean ± SD. (**C**) Microscopic observations of CA-III expressed in MΦ incubated with anti-CAIII Ab for 0, 0.5, 1, and 2 h by immunofluorescence. CA-III (red) and nuclei (blue) were stained according to previously reported methods [11]. High power magnification was used to acquire all images (×1000). White scale bar = 10 μm. CA-III: carbonic anhydrase III; MΦ: peritoneal macrophages; FICS: fluorescent immunochemical staining.

Western blotting showed that the expression of CA-III decreased to some extent in MΦ treated with anti-CAIII Ab (Figure 8A and Figure S5). Furthermore, anti-CAIII Ab treatment resulted in suppression of CA-III mRNA in MΦ (Figure 8B). Immunofluorescence staining showed that CA-III in MΦ gradually disappeared after treatment with the anti-CAIII Ab (Figure 8C). 

### 3.8. Evaluation of Effects of CA-III on Cytokine Secretion from MΦ

**Figure 9:**
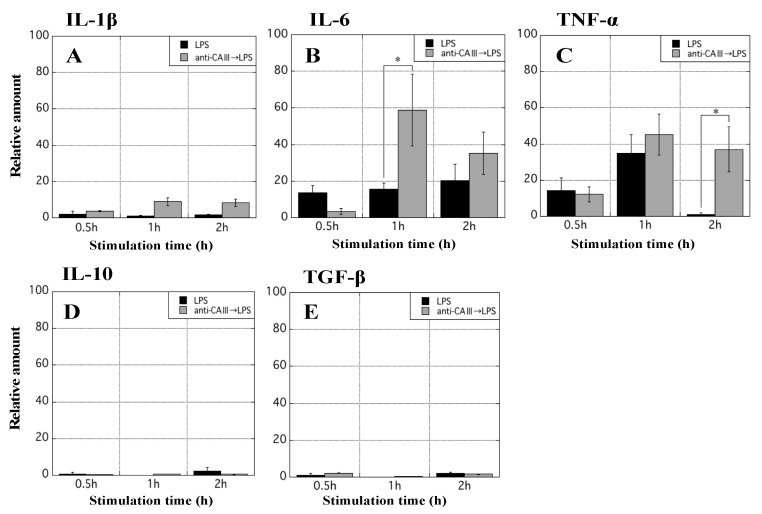
Measurement of inflammatory cytokine mRNA in rat MΦ stimulated with LPS. Real-time PCR was performed to quantitatively measure the mRNA of IL-1β (**A**), IL-6 (**B**), TNF-α (**C**), IL-10 (**D**), and TGF-β (**E**) in MΦ stimulated with LPS. The X-axis indicates the stimulation time (0.5, 1, and 2 h), while the Y-axis shows the relative amount of cytokine mRNA. Black bars indicate the relative amount of each cytokine mRNA in MΦ simply stimulated with LPS, while gray bars indicate the relative amount of each cytokine mRNA in MΦ that were pretreated with anti-CAIII Ab for 1 h followed by stimulation with LPS. Data are mean ± SD. * *p* < 0.05. CA-III: carbonic anhydrase III; LPS: lipopolysaccharide; MΦ: peritoneal macrophages; IL: interleukin; TNF: tumor necrosis factor; TGF: transforming growth factor.

Five cytokines in active MΦ treated with/without anti-CAIII were measured. In MΦ stimulated with LPS, the mRNA expression levels of IL-6 and TNF-α were upregulated (Figure 9A). MΦ were pretreated with anti-CAIII Ab for 1 h and subsequently stimulated with LPS. The mRNA expression of inflammatory cytokines, particularly IL-6 and TNF-α increased more in these MΦ than in those stimulated with LPS alone (Figure 9B). The loss of CA-III in MΦ appears to have contributed to an increase in the secretion of inflammatory cytokines and the development of inflammation. 

## 4. Discussion

UC is an intractable disease specified by the Ministry of Health in Japan, and the mechanism underlying its onset remains unclear. In this context, the lack of therapeutic targets may be the strongest and most significant hurdle to the establishment of a treatment technique for this disease in the field of clinical medicine. In addition, the continuous increase in the number of UC patients is currently a concern worldwide.

UC reportedly occurs in the rectal region of the large colon; however, its origin remains unclear. The purpose of the present study was to identify novel biomarkers and therapeutic targets for UC using an experimental animal model. First, changes in the levels of some proteins in the rectal tissue of UCR were investigated, and the expression of some components was significantly different between WT and UCR rats. Among them, the most noticeable protein was CA-III, which encouraged us to proceed with scientific research to determine the origin of UC. The intracellular concentration of CA-III was significantly decreased in MΦ, although the reason for this is unclear. We previously reported the possibility that S100 proteins are deeply involved in the onset and aggravation of UC and that immunological assessments of the balance between S100A8 and S100A9 may be used to establish the current state of MΦ in the gastrointestinal tract [12]. Furthermore, an in vivo study using transgenic rats that systemically express S100A8 demonstrated that S100A8 is an anti-inflammatory protein [13]. The immunological functions of S100 proteins have been demonstrated not only in UC but also in rheumatoid arthritis [27,28] and inflammatory skin diseases [29]. Because S100 proteins are not always specific biomarkers for UC, unknown proteins in the large intestine of UCR were analyzed to attempt to discover more specific biomarkers. A comparison of protein components in the rectal region between healthy individuals and patients with severely inflamed UC is the most effective strategy. However, it was not possible to perform a comparative study using human samples. The in vivo study reported herein, which used experimental UC, is a simple and effective strategy, although the experimental UC induced by administration of 5% DSS essentially differs from UC in humans. Experimental UC is generally induced by the administration of 5% DSS in rats (Figure 2A,B) because DSS is directly toxic to the colonic epithelium and can lead to reproducible acute colonic inflammation [30]. Furthermore, pathological findings of DSS-induced colitis are similar to those of UC in humans. Although the onset and aggravation of UC in humans have been suggested to involve environmental and genetic factors [4], experimental colitis induced with 5% DSS is a useful animal model for examining the innate immune mechanisms of UC. Indeed, the most severe disease in the rectal region of UCR was observed on Day 10 (Figure 2C), suggesting that novel biomarkers could be identified in the tissue. The protein components in the rectal tissue in WT and UCR on Day 10 were separated by GFC and SDS-PAGE. It was difficult to cleanly separate the proteins because a short gel filtration column (a PD-10 column embedded with 8.3 mL Sephadex G-25) was used in this assay.

Nevertheless, the column enabled us to compare, to some extent, the results obtained by SDS-PAGE using the rectal proteins and to select protein bands with different expression levels between WT and UCR. The levels of eight protein bands (B1–B8) appeared to decrease in the rectal region of UCR, while the levels of three protein bands (B9–B11) increased (Figure 3). Six protein bands in the gel were correctly identified using mass spectrometry, but six bands could not be identified (Figure 4 and Table 3). Although some proteins such as fructose-bisphosphate aldolase A (P5) and transgelin (P10) were of interest, we focused on CA-III (P6) in the present study. 

Carbonic anhydrases (CAs) are zinc-containing enzymes that reversibly catalyze the hydration of carbon dioxide in the body to maintain homeostasis [31]. Previous studies have reported the involvement of CA family proteins in specific diseases, such as CA-VI in Sjögren’s syndrome [32] and CA-IX in renal cell carcinoma [33]. Reductions in CA-I have been reported in the colonic mucosa of patients with active UC [34]. Furthermore, recent findings from animal experiments revealed that epitope peptides of CA-I may contribute to the attenuation of inflammation in the colon by inducing anti-inflammatory cytokines such as IL-10 and TGF-β [35]. Similarly, CA-IV was significantly downregulated in colonic epithelial cells from experimental acute and chronic UC [36]. As discussed above, the potential of some CA family proteins as biomarkers and therapeutic targets has been reported. However, in this study, we’ve focused on the cause-and-effect relationship between experimental UC and CA-III fluctuations in rectum and serum for the first time in the world. 

Immunohistochemical staining showed that the mucosal epithelium in the rectal region of WT abundantly expressed CA-III, whereas its amount decreased with the aggravation of inflammation in the rectum of UCR (Figure 5A). Many immune cells in the colon, such as neutrophils and MΦ, accumulate in and infiltrate the mucosal epithelium. The present study suggests that CA-III contributes to the regulation of the immunological functions of MΦ. The usefulness of serum CA-III level as a biomarker for experimental UC is shown in Figure 5B. We previously investigated the relationship between serum S100A8/A9 and DAI scores in patients with UC; however, the R-value did not exceed 0.342 [16]. The relationship between serum complement C3 or α_2_-macrogulobulin concentrations and DAI scores was also evaluated, with R-values of 0.581 and 0.474, respectively [17]. The R-values between serum CA-III and the DAI and HIS scores were 0.637 and 0.835, respectively (Figure 5C,D). These results are superior to those of our previous study and strongly suggest that serum CA-III may be applicable to monitor not only the physical condition of UC but also the severity of inflammation in the large intestine. The concentration of CA-III protein and its mRNA decreased in the large intestine, particularly in the rectal region, of UCR (Figure 6), which strongly suggests the potential of CA-III as a useful biomarker and/or therapeutic agent for UC. However, despite extensive research, the function of CA-III remains unclear. The in vitro study to clarify the immunological properties of CA-III in cells using MΦ of WT is a rational and adaptive strategy in this study. 

In that in vitro study, CA-III was abundantly expressed in normal MΦ but to a lesser extent in MΦ stimulated with LPS (Figure 7). In UC, the nuclear factor-kappa B (NF-κB) pathway in immune cells was previously shown to be strongly activated for the exacerbation of inflammation by LPS derived from gut microbiota such as E. coli [37]. Therefore, an in vitro assay was performed to replicate the conditions of MΦ in the large intestine of UCR. These results suggest that CA-III in immune cells is involved in the decrease in the protein levels in the colon of UCR. The immunological functions of MΦ with lower intracellular levels of CA-III were also evaluated; the levels of CA-III and its mRNA were markedly decreased by treatment with anti-CAIII Ab (Figure 8). A previous study indicated that serum anti-CAII antibodies were elevated in patients with UC [38]. Thus, the suppression of CA proteins by specific antibodies may be involved in the aggravation of UC. To test this hypothesis, we assessed the mRNA expression of five cytokines in MΦ stimulated with LPS after treatment with an anti-CAIII Ab. The mRNA expression of IL-6 and TNF-α was more strongly upregulated in LPS-stimulated MΦ treated with anti-CAIII Ab than in antibody-untreated MΦ (Figure 9). Based on this result, we predicted that intracellular CA-III may function as an immune regulator, suppressing the expression of inflammatory cytokines in the cells. It was assumed that the amount of CA-III in each cell type, such as mucosal epithelial cells in the rectum and MΦ, decreases in the inflammatory region. Based on this hypothesis, if CA-III is successfully delivered to the inflammatory region, it may improve the pathological condition in the inflamed region of the large colon of UCR. However, to clinically apply CA-III as a therapeutic agent for UC, further studies are needed to elucidate the underlying mechanisms. We hypothesized that CA-III may play an important role in a specific pathway that exacerbates inflammation, such as the NF-κB and mitogen-activated protein kinase pathways. As limited information hampers efforts in the clinical applications of CA-III, further studies may be needed.

The measurement of serum CA-III levels is useful for diagnosing UC in early stages. In the future, we intend to measure serum CA-III concentrations in patients with mild-to-severe UC. The clinical application of CA-III makes it challenging to clarify the possible use of CA-III as a therapeutic agent for UC. To confirm its pharmacological effects in fundamental research, a large amount of purified CA-III may be needed to treat the inflamed rectal tissues of UCR. 

## 5. Conclusions

We found that CA-III reflects the severity of experimental colitis. Thus, serum CA-III level may be a useful biomarker for experimental UC. In addition, CA-III in the colonic mucosa and MΦ significantly suppressed the secretion of inflammatory cytokines and protected against aggravation of experimental UC. Therefore, CA-III may be an important tool for investigating the causes of UC.

## Figures and Tables

**Table 1:** Criteria used for disease activity index (DAI) scoring.

DAI Scores			
Scores	Weight Loss (%)	Stool Consistency	Occult/Gross Bleeding
**0**	None 1–5	Normal	Normal
**1**			
**2**	6–10	Loose stool	Occult bleeding
**3**	11–20		
**4**	>20	Diarrhea	Gross bleeding

**Table 2:** Criteria used for histological (HIS) scoring.

Scores	HIS Scores
**0**	Normal colonic mucosa
**1**	Goblet cell depletion on crypts of less than 1/3
**2**	Goblet cell depletion on crypts ranging between 1/3 to 2/3
**3**	Mucosal erosion (partial loss of the epithelium with the basement membrane left intact)
**4**	Mucosal erosion or ulcers (extensive loss of the epithelium including the basement membrane) with the significant infiltration of inflammatory cells

**Table 3:** Identification of protein components via the NCBI database.

Band	Sample	Analyzed Results	Score
B1	P1	Transient receptor potential cation Channel subfamily M member 6 isoform X1	103
B4	P4	beta-enolase	89
B5	P5	Fructose-bisphosphate aldolase A	82
B6	P6	Carbonic anhydrase III	102
B7	P7-1	Hemoglobin, alpha 2	150
	P7-2	rCG39881, isoform CRA_a	120
B10	P10	Transgelin	123

## Data Availability

The data presented in this study are available in this article.

## Supplementary Materials

The following supporting information can be downloaded at: www.mdpi.com/article/10.3390/biology11040494/s1, Figure S1: The original SDS-PAGE figures regarding fractionation and separation of rectal proteins in WT rats (Figure 3A), Figure S2: The original SDS-PAGE figures regarding fractionation and separation of rectal proteins in UCR group (Figure 3B), Figure S3: The original Western blotting figures of CA-III and β-action detected in the colon of WT rats and UCR group on Day 10 (Figure 6B), Figure S4: The original Western blotting figures of CA-III and β-action detected in MΦ (Figure 7A), Figure S5: The original Western blotting figures of CA-III and β-action detected in MΦ (Figure 8A).
